# Development and validation of an analytical method for the determination of direct oral anticoagulants (DOAC) and the direct thrombin-inhibitor argatroban by HPLC–MS/MS

**DOI:** 10.1007/s11239-021-02596-z

**Published:** 2021-11-11

**Authors:** Lea Brückner, Jan Beyer-Westendorf, Oliver Tiebel, Jörg Pietsch

**Affiliations:** 1 Hochschule Für Technik Und Wirtschaft, Friedrich-List-Platz 1, 01069 Dresden, Germany; 2 Division Hematology, Department of Medicine 1, Universitätsklinikum Dresden, Fetscherstr. 74, 01307 Dresden, Germany; 3 Institute of Clinical Chemistry and Laboratory Medicine, Universitätsklinikum Dresden, Fetscherstr. 74, 01307 Dresden, Germany; 4 Institute of Legal Medicine, Technische Universität Dresden, Fetscherstr. 74, 01307 Dresden, Germany

**Keywords:** Argatroban, Direct thrombin-inhibitor, Direct oral anticoagulants, DOAC, High performance liquid chromatography—tandem mass-spectrometry (HPLC–MS/MS)

## Abstract

Since direct oral anticoagulants (DOAC) are administered frequently to an elderly, co-morbid population, medical emergencies including trauma, acute bleeding or organ failure are not uncommon. In these situations, the type, dosage or the time of last intake of anticoagulants is often unknown and single substance analysis by functional tests is only possible if the substance contained in the sample is known. A reliable and validated toxicology screen of DOAC and argatroban would be helpful inform not only attending physicians in the emergency department but also law enforcement and courts of justice. After precipitation with acetone, HPLC separation was achieved on a Phenomenex Luna Pentafluorophenyl Colum using acetonitrile–water (90:10, v/v) as mobile phase system. Detection was performed using a 3200 Q Trap mass spectrometer (AB Sciex). For analysis MRM Scans (MS/MS) with positive ionization were chosen. The method was validated for blank serum as the matrix of choice. Limits of detection are between 0.5 and 1.0 ng/mL, limits of quantification are between 1.9 and 3.6 ng/mL and recoveries are above 60%. The applicability of the method was demonstrated by the determination of DOAC in body fluids from forensic cases and in therapeutic drug monitoring. The rapid simultaneous detection and quantification of apixaban, argatroban, dabigatran etexilate, dabigatran, edoxaban and rivaroxaban in body fluids by HPLC–MS/MS closes an important gap in emergency toxicology.

## Highlights

- Up to now, a few HPLC methods determining only a subset of DOAC exist.
- A selective and sensitive HPLC–MS/MS method after precipitation with acetone was developed.
- Four DOAC and argatroban are simultaneously determined in the trace range in body fluids.
- The method is appropriate for therapeutic drug monitoring and intoxication cases.

## Introduction

Over the last 15 years, numerous new anticoagulants have been developed and introduced in clinical care, including the parenteral direct thrombin inhibitor argatroban [1] and direct oral anticoagulants (DOAC; alternatively called non-vitamin-K dependent oral anticoagulants or NOAC) [2–5]. The latter group of drugs was developed as alternatives to low molecular heparins and vitamin K antagonists (VKA) for therapeutic anticoagulation and constitutes of direct oral factor Xa inhibitors (apixaban, edoxaban, rivaroxaban) and the direct oral thrombin inhibitor (dabigatran etexilate).

The DOAC offer some advantages over their predecessors, including oral intake, a fixed dosing (based on a predictable dose–response relationship) and, compared to VKA, a reduced risk for drug-food and drug-drug interactions. For routine use of DOAC, no therapy monitoring is necessary [6, 7]. If needed, functional assays are used to determine DOAC. These assays measure the change of the thromboplastin time for direct factor Xa inhibitors and the change of the activated partial thromboplastin time (aPTT) for direct thrombin inhibitors. For quantification, anti-factor Xa activity is measured for factor Xa inhibitors and modified thrombin time for direct thrombin inhibitors [8].

However, since anticoagulants are administered frequently to an elderly, co-morbid population, medical emergencies including trauma, acute bleeding or organ failure are not uncommon. In these situations, the type, dosage or the time of last intake of anticoagulants is often unknown and single substance analysis by functional tests is only possible if the substance contained in the sample is known. Furthermore, accidental, suicidal or criminal DOAC intoxications may occur for which a reliable and validated toxicology screen would be helpful inform not only attending physicians in the emergency department (ED) but also law enforcement and courts of justice.

Up to now, only a few methods for the qualitative and quantitative determination of DOAC exist. HPLC–MS/MS methods are most commonly used [9–12] but ultraviolet–visible spectrophotometric methods are also available [13–17]. However, most of these methods determine only a subset of these drugs, which is clearly a downside in an emergency toxicology screening setting. To overcome this limitation, an HPLC–MS/MS method for the simultaneous determination of five oral or parenteral new anticoagulants has been developed and validated.

## Materials and methods

### Chemicals and reagents

Acetonitrile and water, both of HPLC-grade, ammonium formate, ethanol and formic acid were purchased from VWR International (Fontenay-sous-Bois, France). Apixaban, edoxaban and rivaroxaban were supplied by the Cayman Chemical Company (Ann Arbor, USA). Dabigatran etexilate and argatroban monohydrate were purchased from Sigma Aldrich (St. Louis, USA) and dabigatran from biosynth/carbosynth (Thal, Switzerland). Hydrochloric acid was supplied from Merck (Darmstadt, Germany) and acetone was supplied from Fisher Scientific (Loughborough, UK). Methanol was obtained from PanReac AppliChem (Darmstadt, Germany). Apixaban-13C-d_3_ (Cayman Chemical Company, Ann Arbor, USA) was used as internal standard (IS). The blank serum for method validation was obtained from the German Red Cross blood donation in Dresden, Germany.

Quantification by functional testing was performed applying STA Liquid Anti-Xa Kit with drug specific calibration on a STA R Max3 analyzer (reagents, calibrators & analyzer: STAGO Deutschland GmbH, Düsseldorf, Germany).

### Preparation of calibration standards

Standard stock solutions of the DOAC and of argatroban were individually prepared in methanol at 1.0 mg/mL, respectively. The five stock solutions were combined in methanol and serially diluted with methanol to produce combined standard working solutions at concentrations of 100, 10, 1.0, 0.10, 0.01, 0.001 µg/mL. The IS working solution was diluted with methanol to 10 µg/mL for apixaban-13C-d_3_. Calibration samples were freshly prepared by spiking 10, 5.0 or 2.5 µL of the working solutions into blank serum to give final concentrations between 0.50 and 1000 ng/mL. All of the solutions were stored at − 20 °C.

### Sample extraction

For calibration the DOAC, argatroban and IS stock solutions were added to a tube filled with 500 µL of blank serum, respectively, followed by adding of 100 µL hydrochloric acid and 1.0 mL acetone. The mixture was then vortex-mixed for two minutes and centrifuged at 14,000 rpm for five minutes. The supernatant solution of the precipitation was transferred into another tube and evaporated to dryness under a gentle stream of nitrogen at 50 °C. Finally, the dried residue was dissolved in 100 µL of the particular mobile phase mixture, vortex-mixed and centrifuged. The supernatant was then transferred to an autosampler vial and 20 µL were injected for analysis. The same procedure was applied to the body fluids serum, urine and stomach content (using 100 µL of stomach content).

### Instrumentation

HPLC–MS/MS analysis was performed using a 1260 infinity quaternary LC system (Agilent Technologies), equipped with a Phenomenex Luna Pentafluorophenyl Column (length: 150 mm; internal diameter: 2 mm; particle size: 5 µm), and a 3200 Q Trap (AB Sciex) mass spectrometer. The separation was obtained with the mobile phases water/ammonium formate (2 mM)/formic acid (0.2%) mixture (solvent A) and acetonitrile/ammonium formate (2 mM)/formic acid (0.2%) mixture (solvent B). Gradient employed went from initial 90:10 (A:B) to 10:90 (A:B) in 10 min with a flow rate of 0.5 mL/min. This was held for 5 min and a flow-rate of 1 mL/min was applied. Then the gradient went from 10:90 (A:B) at 15 min back to 90:10 (A:B) at 16 min using a flow rate of 1 mL/min. For the last 6 min of the measurement the gradient was not changed and a flow rate of 0.5 mL/min was used. The system was operated at room temperature (20 °C). The detection was performed after electrospray ionization using MRM scans in positive ion mode. Dwell time was optimized for each mass transition. The source temperature was maintained at 500 °C and the spray voltage was set at 5000 V. Ion source gas 1, ion source gas 2 and curtain gas were set at 45, 60 and 20, respectively. The MS-parameters declustering potential (DP), collision energy (CE), cell exit potential (CXP) were optimized by supplying the MS with standard stock solutions via the HPLC and by varying the MS parameters. The two most intensively MRM transitions of each molecule were determined.

### Validation of the method in serum

Precision and accuracy, stability, linearity and recovery were analysed following Peters et al. [18] using Valistat 2.0 software (ARVECON GmbH, Germany) [19].

#### Specificity and selectivity

To evaluate potential endogenous interferences in human serum, a comparison of five blank serum samples and five serum samples spiked with the IS was performed based on the responses at the retention times of the analytes.

#### Precision and accuracy

The precision and accuracy were determined by the extraction and analysis of two replicate quality control samples (QC) at low and high concentration (QC_low_: 20 ng/mL and QC_high_: 100 ng/mL) on eight consecutive days. The accuracy is the deviation of the mean concentration and the measured concentration. It was required to be within ± 15%. Precision was expressed as percentage relative standard deviation (RSD) and was required not to exceed 15% of the corresponding standard solutions.

#### Stability

The stability of DOAC and argatroban in prepared samples during the measurement was assessed by six measurements of QC samples at low and high concentration (QC_low_: 20 ng/mL, QC_high_: 100 ng/mL). The measurements are always taken from the same vial for each concentration. There are 66 min between two measurements of the same concentration.

#### Linearity, limit of detection (LOD) and limit of quantification (LOQ)

Linearity, LOD and LOQ were assessed by plotting the peak area of the analyte vs. analyte concentration (ten calibration points from blank samples spiked with the standards: 0.5, 1.0, 5.0, 10, 20, 50, 100, 200, 500, 1000 ng/mL for linearity and 0.02, 0.1, 0.2, 0.5, 1.0, 2.0, 5.0, 10, 20, 50 for LOD and LOQ) in fresh prepared serum calibrators. Calibration curves were analyzed by weighted linear regression of the peak area of the analyte vs. nominal concentrations.

#### Extraction recovery

The extraction recovery is determined at two concentration levels (QC_low_: 100 ng/mL, QC_high_: 500 ng/mL). The percentage of the mean peak areas of the extracted samples is calculated from the peak areas of the unprocessed samples of the same concentration.

### Analysis of clinical and forensic samples

The practical suitability of the validated method is to be tested by measuring real samples. The Institute of Legal Medicine Dresden, Germany, provided the matrices urine, stomach content and venous blood of three deceased persons. The decedents were all receiving DOAC medication (rivaroxaban n = 2; apixaban n = 1) but did not die from intoxication. Urine samples had to be diluted before extraction (1:10 and 1:50) in order to get concentrations within the calibration range. In addition, six apixaban plasma samples, three dabigatran etexilate plasma samples, two edoxaban plasma samples and five rivaroxaban plasma samples from clinical routine patients undergoing of therapeutic drug monitoring (TDM), were provided by the Department for Clinical Chemistry and Laboratory Medicine of the Universitätsklinikum Dresden, Germany.

## Results

### Optimization of HPLC–MS/MS method

**Fig. 1:**
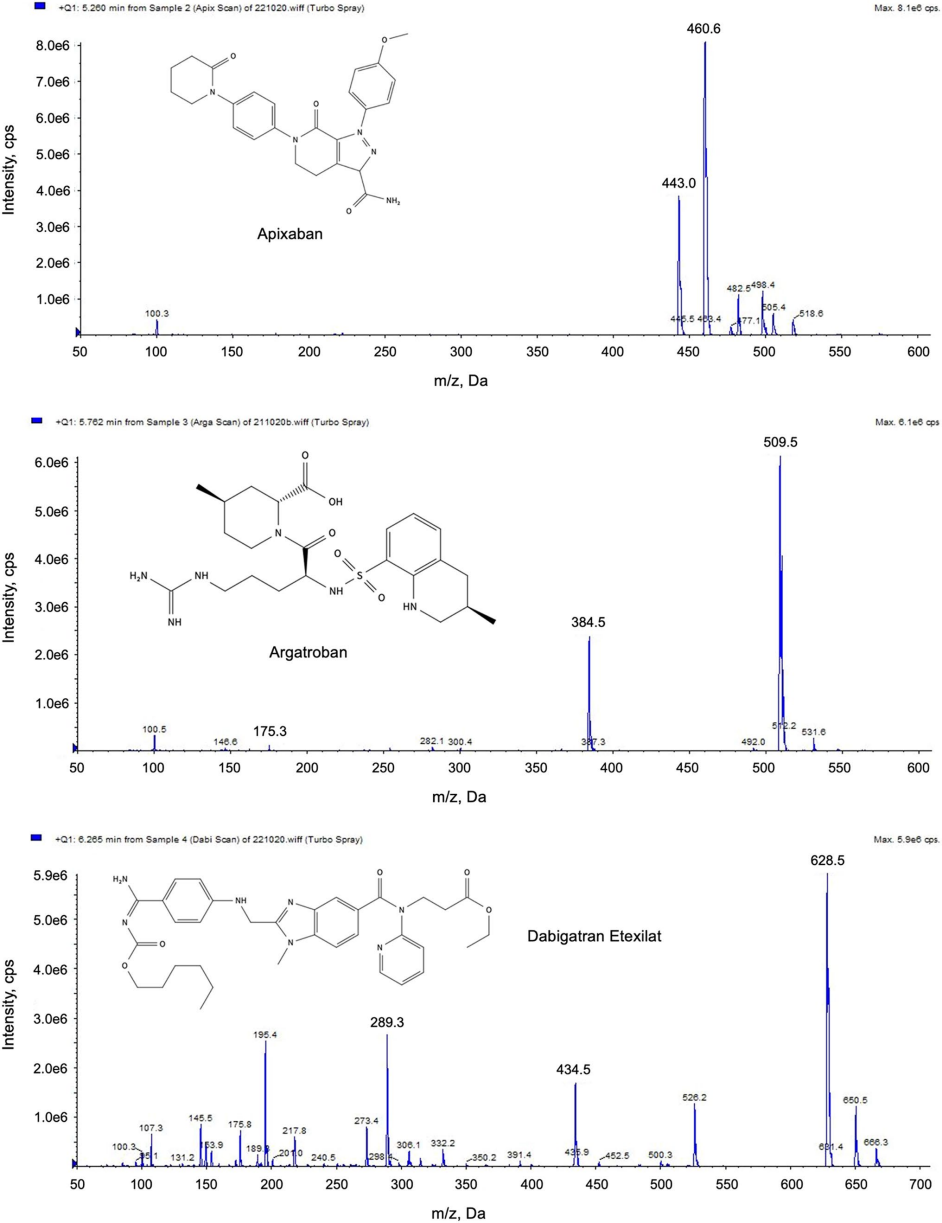

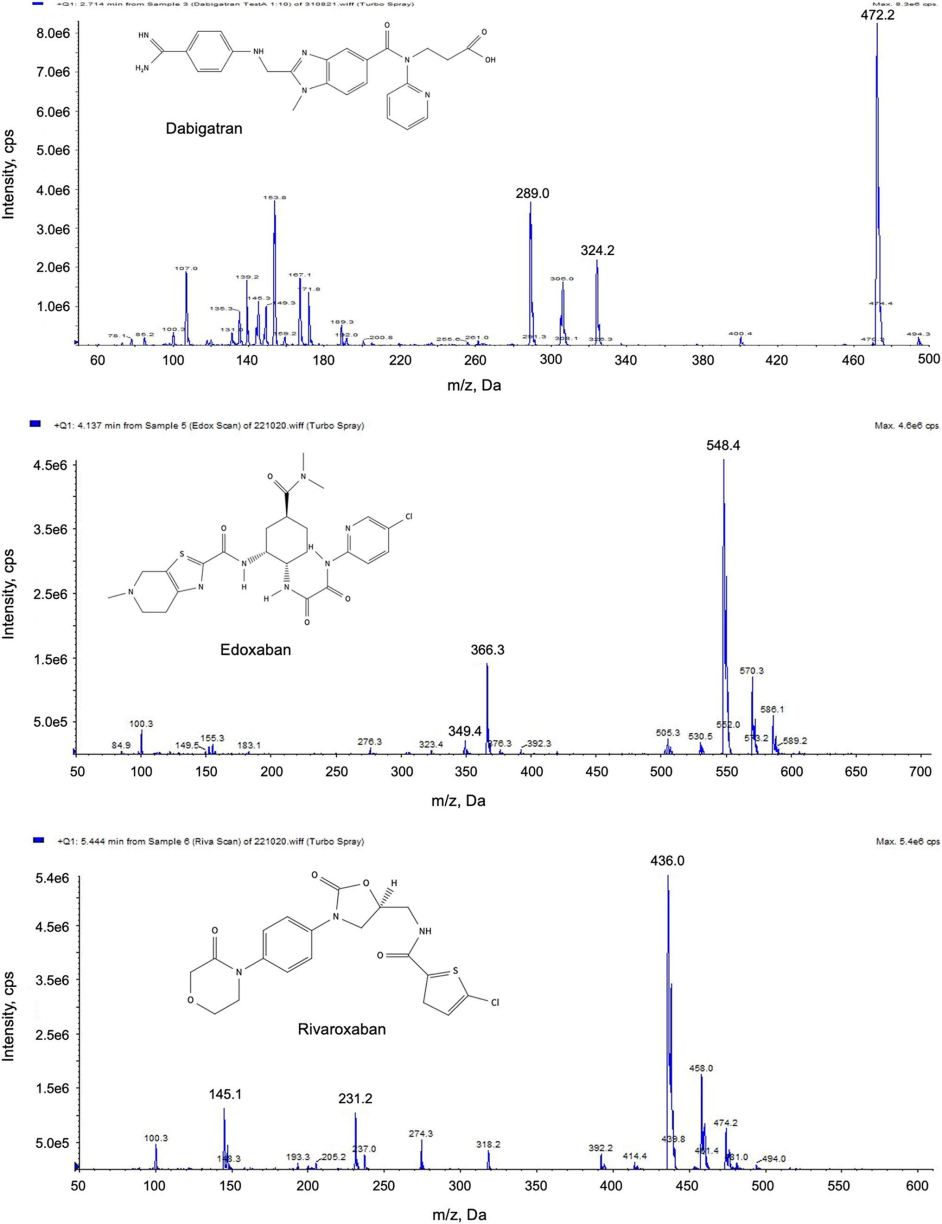
Full-scan product ion spectra of the selected DOAC

Ionization of the compounds was performed by electrospray ionization. To optimize the MS conditions, the analytes and the IS were tested in positive ionization as Q1 scan and in the MRM modus. Substance-specific MRM transitions were derived from the most intense signals in the Q1 scan (Fig. 1).

Based on the mass spectra shown in Fig. 1 quantifier and qualifier ions were defined for each anticoagulant. The optimized MS parameters for the detection of the selected anticoagulant and the IS apixaban-13C-d_3_ are summarized in Table 1.

**Table 1:** Optimized chromatographic and MS parameters (ESI +)

Compound	Retention time (min)		Parent ion (m/z)	Daughter ion (m/z)	DP (V)	CE (V)	CXP (V)
Apixaban	5.30	Quantifier	460.6	443.1	50	20	4
		Qualifier	460.6	77.0	50	64	4
Edoxaban	4.20	Quantifier	548.4	366.3	40	35	4
		Qualifier	548.4	349.4	50	35	4
Rivaroxaban	5.45	Quantifier	436.0	145.1	50	35	4
		Qualifier	436.0	231.2	50	28	4
Argatroban	5.80	Quantifier	509.5	384.5	50	20	4
		Qualifier	509.5	175.3	50	65	4
Dabigatran etexilate	6.30	Quantifier	628.5	289.4	40	50	4
		Qualifier	628.5	434.2	40	35	6
Dabigatran	2.40	Quantifier	472.2	289.0	40	50	4
		Qualifier	472.2	324.2	40	50	4
Apixaban-13C-d_3_	5.33	Quantifier	463.7	447.2	56	52	4

*DP* declustering potential, *CE* collision energy, *CXP* cell exit potential

**Fig. 2:**
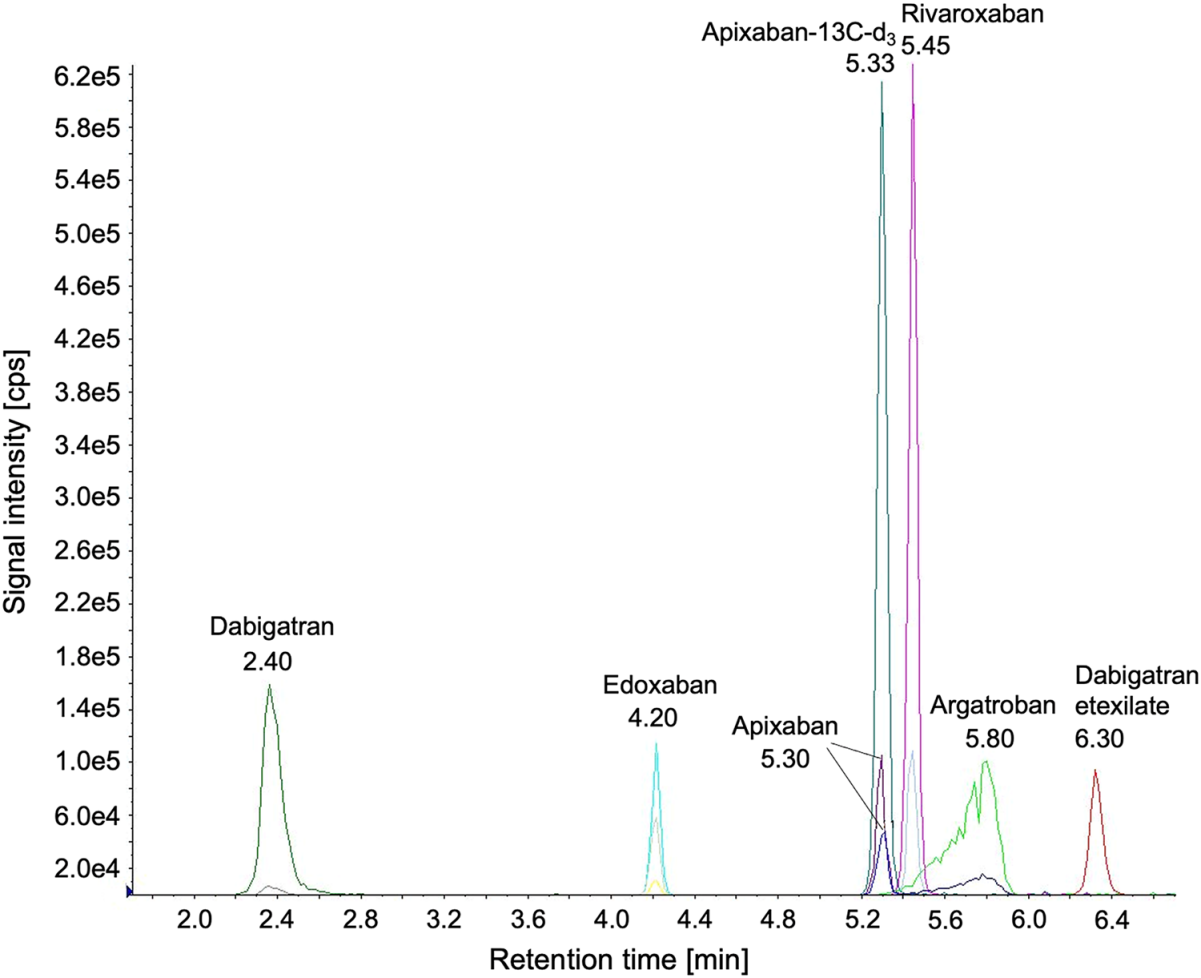
Chromatogram of the final HPLC–MS/MS (ESI+) method

The chromatographic separation of the selected DOAC is shown in the HPLC-(ESI +)-MS/MS chromatogram in Fig. 2.

### Method validation

#### Specificity and selectivity

No peaks caused by disturbing substances could be detected in the zero and blank samples at the same retention times as the analytes.

#### Precision and accuracy

Table 2 summarizes precision and accuracy of the five analytes. As seen the substances comply with the usual validation criteria: up to 15% (20% at low concentrations) for accuracy and precision.

**Table 2:** Accuracy and precision of the developed HPLC–MS/MS method (inter-day variations)

	Mean ± SD (ng/mL)	Accuracy (%)	Precision (RSD %)
20 ng/mL			
Apixaban	18.3 ± 1.3	− 8.3	7.6
Edoxaban	17.9 ± 2.6	− 10.3	14.9
Rivaroxaban	20.5 ± 3.0	2.7	14.9
Argatroban	20.3 ± 2.1	1.8	10.6
Dabigatran etexilate	19.1 ± 2.4	− 4.4	12.6
Dabigatran	21.1 ± 2.6	5.8	12.6
100 ng/mL			
Apixaban	97.7 ± 6.1	− 2.2	6.2
Edoxaban	105.1 ± 7.7	5.1	7.3
Rivaroxaban	100.9 ± 10.8	1.0	10.7
Argatroban	104.3 ± 8.4	4.3	8.1
Dabigatran etexilate	103.2 ± 12.9	3.3	12.5
Dabigatran	101.4 ± 15.2	1.4	15.0

*RSD* relative standard deviation

#### Stability

Chemical stability of apixaban, argatroban, dabigatran etexilate, edoxaban, dabigatran and rivaroxaban was confirmed in processed samples for a period of 374 min.

#### Linearity, limit of detection (LOD) and limit of quantification (LOQ)

The method validation parameters Linearity, LOD, LOQ and extraction recovery in serum are reported in Table 3 and the HPLC–MS/MS demonstrated good sensitivity (LOD: 0.31 to 1.04 ng/mL; LOQ: 1.11 to 3.57 ng/mL) with extraction yields ranging from 62 to 93%.

**Table 3:** Method validation parameters in serum

Compound	Linearity (ng/mL)	LOD (ng/mL)	LOQ (ng/mL)	Extraction recovery (%)	
				100 ng/mL	500 ng/mL
Apixaban	0.5–100	0.54	1.89	65.0	66.0
Edoxaban	1.0–500	1.04	3.57	93.0	86.0
Rivaroxaban	0.3–500	0.31	1.11	62.0	61.0
Argatroban	1.0–1000	0.95	3.27	79.0	69.0
Dabigatran etexilate	0.9–1000	0.94	3.23	74.0	78.0
Dabigatran	1.9–500	0.55	1.96	71.1	79.7

*LOD* limit of detection, *LOQ* limit of quantification

### Analysis of clinical and forensic samples

**Fig. 3:**
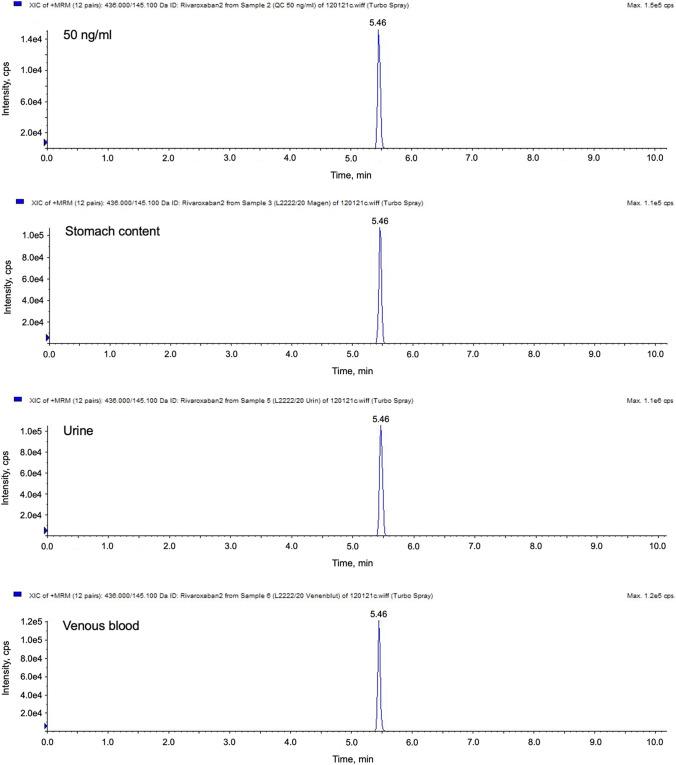
Extracted ion chromatograms for rivaroxaban (m/z 145.1) in a calibration standard (50 ng/mL) as well as stomach content, urine and venous blood of the deceased person #1

Figure 3 and Table 4 summarize the determined concentrations of rivaroxaban respectively apixaban in body fluids of three deceased persons. Rivaroxaban was detected in the deceased person #1 and #2 and apixaban in the deceased person #3.

**Table 4:** Concentrations of the detected analytes in deceased persons

	Concentrations of the compounds (ng/mL)	
	Apixaban	Rivaroxaban
Deceased person #1		
Urine	< LOD	7160
Stomach content	< LOD	1315
Venous blood	< LOD	88
Deceased person #2		
Urine	< LOD	659
Stomach content	< LOD	40
Venous blood	< LOD	301
Deceased person #3		
Urine	738	< LOD
Stomach content	123	< LOD
Venous blood	76	< LOD

The concentration of rivaroxaban measured in the venous blood of the deceased person #1 and the concentration of apixaban measured in the venous blood of the deceased person #3 are within the therapeutic range. Concentrations measured in the venous blood of the deceased person #2 are outside the therapeutic range of rivaroxaban. According to case studies [20], this concentration is to be classified in the toxic range.

Table 5 shows the results of the samples provided by the IKL. The concentrations determined by the HPLC–MS/MS method and the concentrations determined by the IKL by functional tests were compared and, in both tests, consistently fell in the respective therapeutic ranges of the DOAC.

**Table 5:** Comparison of concentrations determined by HPLC–MS/MS and by functional tests

Compound	Sample #	HPLC–MS/MS (ng/mL)	DOAC concentration via anti-FXa-activity-assay (ng/mL)
Apixaban	1	177	156
	2	221	211
	3	318	303
	4	120	73
	5	< LOD	337
	6	199	168
Edoxaban	1	8	23
	2	139	148
Rivaroxaban	1	84	137
	2	221	385
	3	35	44
	4	228	493
	5	106	255
Dabigatran	1	253	> 500
	2	196	Positive
	3	68	Positive

Rivaroxaban was detected and quantified in (falsely labeled) apixaban sample #5 due to the fact that, at the time of IKL analyses, clinical information was misleading, falsely indicating an intake of apixaban instead of the actual intake of rivaroxaban. The analyte dabigatran could be detected in any of the three samples by the HPLC–MS/MS method, confirming the positive results of the functional tests.

## Discussion

The widespread clinical use of potent and potentially dangerous anticoagulant drugs raises the need for rapid qualitative or, even better, quantitative testing of anticoagulant activity in case or emergency situations. For instance, clinical management of acute bleeding, major trauma requiring urgent surgery or thrombolytic treatment of acute thromboembolism such as cardioembolic stroke may depend on the intensity of anticoagulation activity at the time of presentation in the emergency department. Available tests are limited by 24/7 availability or the lack of a quantification of the DOAC. Most importantly, such tests usually require the information, which anticoagulant was taken so that the correct assay or calibration curve can be utilized. In cases where such information is lacking, a screening assay that combines specific and sensitive detection of several anticoagulant drugs could be helpful, although the HPLC–MS/MS method presented here may not resolve this since a widespread 24/7 availability may also remain limited to tertiary care centers. However, the following two real-life cases from our clinical routine illustrate the importance of such a toxicology screening tool.

The first case was a 68 year old female patient admitted to our intensive care unit with progressive decrease of consciousness, hypotension, massive diarrhea and diffuse mucosal bleeding. The husband described unspecific discomfort and weakness during the course of the day and confirmed chronic treatment with dabigatran 110 mg twice daily for stroke prevention in atrial fibrillation. Laboratory tests indicated massively deranged coagulation (thromboplastin time and activated partial thromboplastin time not measurable; INR > 10, fibrinogen < 0.4 g/L) with only moderately elevated inflammation markers, normal blood counts and normal values for antithrombin and d-dimer. Serology confirmed only minor abnormalities in renal and liver function tests. Catastrophic antiphospholipid syndrome, acquired hemophilia and disseminated intravascular coagulation were taken into account but considered implausible in the clinical context and dedicated laboratory assays did not detect confirming antibodies. Immediately, dabigatran overdosing—suicidally or accidentally – was considered but the patient strictly denied overdosing. Despite this, 5 g of the specific dabigatran antidote idarucizumab (Praxbind®) were administered but did neither improve the bleeding nor the coagulation test abnormalities. In this setting, dabigatran overdosing would normally seem very unlikely but when we quantified dabigatran plasma concentration the result of 6200 ng/ml dabigatran etexilate and 253 ng/mL dabigatran (Table 5) despite specific antidot reversal confirmed massive intoxication (therapeutic range for 110 mg BID 85–200 ng/mL), obviously overriding the effects of the specific and potent antidote idarucizumab. High-turnover dialysis was initiated and supportive treatment consisted of 2 g fibronogen, 2 units of fresh frozen plasma and 2000 units of activated prothrombin complex concentrate. Anticoagulation lab tests as well as clinical condition and bleeding entirely normalized within 3 days and the patient restarted dabigatran therapy at discharge for stroke prevention in atrial fibrillation. Law enforcement was involved to further investigate the nature of the massive dabigatran intoxication.

The second case was a 77 year old male patient acutely admitted for open aortic repair following hemodynamic collaps due to ruptured infrarenal aortic aneurysm with extensive retroperitoneal hematoma (total blood loss estimated at 10 L). Before admission, the patient was taking dabigatran 150 mg twice daily for atrial fibrillation. Surgery was performed successfully, with hemostatic measures including 21 units of packed red cells, 20 units of FFP; 4 platelet transfusion units, 4000 IU of prothrombin complex concentrates (PCC) and 6 g fibrinogen. Post-surgery, the patient was transferred to ICU but showed persistently deranged coagulation parameters. Dabigatran overexposure/rebound was considered as a possible explanation but ongoing consumption coagulopathy was at least equally likely. When the patient was treated with 5 g idarucizumab, prothrombin time and activated partial thromboplastin time improved for 12 h after which a significant deterioration was again observed. At this point, disseminated intravascular coagulation was considered as the most likely explanation, because last intake of dabigatran was more than 3 days ago, complete blood exchange occurred from massive intraoperative blood transfusions, and because renal replacement therapy and treatments with PCC and, later, idarucizumab made ongoing dabigatran effects very unlikely. However, when dabigatran quantification was initiated in parallel, supratherapeutic levels of 440 ng/ml dabigatran etexilate and 68 ng/mL dabigatran (therapeutic range for 150 mg BID 117–275 ng/mL) were detected, confirming dabigatran accumulation as a major driver of the patient unstable hemostatic situation despite several days of dabigatran interruption and specific antidote revesal. Therefore, the decision was made to treat with a second course of 5 g idarucizumab which immediately normalized coagulation tests and bleeding symptoms. Unfortunately, the patient did not recover from the initial hemorrhagic collaps and died from multi-organ failure 35 days post admission.

Both cases illustrate that massive DOAC accumulation or intoxication can occur with devastating consequences. The availability of a rapid and reliable toxicology method to detect, differentiate and quantify anticoagulation effects so far has been an unmet clinical need, especially in situations where DOAC exposure and consumption coagulopathy are overlapping scenarios. In both our cases, dabigatran overexposure was considered by the attending physicians as the underlying cause of coagulation derangement and bleeding. However, when specific (idaricizumab) and unspecific (PCC, dialysis) reversal strategies did not improve the situation, dabigatran effects would probably have been rejected as relevant contributing factors. The rapid and validated HPLC–MS/MS quantification method presented here can help to identify anticoagulation effects in emergency situations with severe bleeding and consumption coagulopathy when global coagulation tests, thrombelastography or functional DOAC quantification tests such as diluted prothrombin time (for dabigatran) or functional aXa measurements (for factor Xa inhibitors) do not help to clearly identify the cause of a deranged coagulation. The differentiation between consumption coagulopathy and anticoagulant overexposure is essential for guiding specific treatment decisions. In our cases, the true cause of persistent coagulation derangement, namely the massive and prolonged dabigatran accumulation, could be identified. Similar scenarios can occur for factor Xa inhibitors and event mixed intoxications with more than one DOAC may happen. Of note, clinical information of one of the 5 apixaban sample cases (Table 5) was also misleading: the described intake of apixaban led to a false quantification of aXa activity by using the apixaban calibration curve in a patient actually taking rivaroxaban. As presented in Table 5, such misinformation (potentially misleading the emergency management) can easily be corrected by a MS analysis. Therefore, the development of a rapid simultaneous detection and quantification of apixaban, argatroban, dabigatran etexilate, dabigatran, edoxaban and rivaroxaban in body fluids closes an important gap in emergency toxicology.

## Conclusion

In the present study a HPLC–MS/MS based method for the simultaneous detection and quantification of apixaban, argatroban, dabigatran etexilate, dabigatran, edoxaban and rivaroxaban in body fluids was established. The validated method is specific and sensitive for the four DOAC and argatroban and the extraction protocol showed good extraction yields. Using the method, apixaban, edoxaban, rivaroxaban and dabigatran could be identified and quantified in clinical samples in the therapeutic drug monitoring and in forensic samples.
